# The Vacuolar Ca^2+^ ATPase Pump Pmc1p Is Required for Candida albicans Pathogenesis

**DOI:** 10.1128/mSphere.00715-18

**Published:** 2019-02-06

**Authors:** Arturo Luna-Tapia, Christian DeJarnette, Emily Sansevere, Parker Reitler, Arielle Butts, Kirk E. Hevener, Glen E. Palmer

**Affiliations:** a Department of Clinical Pharmacy and Translational Science, College of Pharmacy, University of Tennessee Health Sciences Center, Memphis, Tennessee, USA; b Department of Molecular Immunology and Biochemistry, College of Graduate Health Sciences, University of Tennessee Health Sciences Center, Memphis, Tennessee, USA; c Department of Pharmaceutical Sciences, College of Pharmacy, University of Tennessee Health Sciences Center, Memphis, Tennessee, USA; Carnegie Mellon University

**Keywords:** *Candida albicans*, Pmc1p, Vcx1p, calcium, pathogenesis, vacuole

## Abstract

Calcium is a critically important secondary messenger of intracellular signal transduction in eukaryotes but must be maintained at low levels in the cytoplasm of resting cells to avoid toxicity. This is achieved by several pumps that actively transport excess cytoplasmic Ca^2+^ out of the cell across the plasma membrane and into other intracellular compartments. In fungi, the vacuole serves as the major storage site for excess Ca^2+^, with two systems actively transporting cytoplasmic calcium ions into the vacuole. The H^+^/Ca^2+^ exchanger, Vcx1p, harnesses the proton-motive force across the vacuolar membrane (generated by the V-ATPase) to drive Ca^2+^ transport, while the P-type ATPase Pmc1p uses ATP hydrolysis to translocate Ca^2+^ into the vacuole. Ca^2+^-dependent signaling is required for the prevalent human fungal pathogen Candida albicans to endure exposure to the azole antifungals and to cause disease within the mammalian host. The purpose of this study was to determine if the Pmc1p or Vcx1p Ca^2+^ pumps are required for C. albicans pathogenicity and if these pumps impact antifungal resistance. Our results indicate that Pmc1p is required by C. albicans to transition from yeast to hyphal growth, to form biofilms *in vitro*, and to cause disease in a mouse model of disseminated infection. Moreover, loss of Pmc1p function appears to enhance C. albicans azole tolerance in a temperature-dependent manner.

**IMPORTANCE** Maintenance of Ca^2+^ homeostasis is important for fungal cells to respond to a multitude of stresses, as well as antifungal treatment, and for virulence in animal models. Here, we demonstrate that a P-type ATPase, Pmc1p, is required for Candida albicans to respond to a variety of stresses, affects azole susceptibility, and is required to sustain tissue invasive hyphal growth and to cause disease in a mouse model of disseminated infection. Defining the mechanisms responsible for maintaining proper Ca^2+^ homeostasis in this important human pathogen can ultimately provide opportunities to devise new chemotherapeutic interventions that dysregulate intracellular signaling and induce Ca^2+^ toxicity.

## Abstract (precis)

Maintenance of Ca^2+^ homeostasis is important for fungal cells to respond to a multitude of stresses, as well as antifungal treatment, and for virulence in animal models. Here, we demonstrate that a P-type ATPase, Pmc1p, is required for Candida albicans to respond to a variety of stresses, affects azole susceptibility, and is required to sustain tissue invasive hyphal growth and to cause disease in a mouse model of disseminated infection. Defining the mechanisms responsible for maintaining proper Ca^2+^ homeostasis in this important human pathogen can ultimately provide opportunities to devise new chemotherapeutic interventions that dysregulate intracellular signaling and induce Ca^2+^ toxicity.

## INTRODUCTION

The fungal vacuole is an acidic intracellular compartment that plays a central role in the maintenance of cellular homeostasis. This includes facilitating the degradation of macromolecules, the storage of crucial metabolites, and the sequestration of potentially toxic substances (1, 2). The vacuole also serves as the major storage site for metal ions, including calcium, a critically important secondary messenger of intracellular signal transduction in eukaryotes. To fulfill its role as a second messenger, Ca^2+^ must be maintained at low levels in the cytoplasm of resting cells (3). This is achieved by several pumps that actively transport excess cytoplasmic Ca^2+^ out of the cell across the plasma membrane or into intracellular compartments, including the endoplasmic reticulum and Golgi apparatus (4). In yeast, more than 90% of the intracellular Ca^2+^ is sequestered in the vacuole (4). Upon appropriate stimulation, Ca^2+^ ions are rapidly released into the cytoplasm from extracellular sources as well as intracellular stores to activate Ca^2+^-dependent proteins such as calmodulin (5, 6). Resting state equilibrium is subsequently restored through calcium efflux, resulting in transient cytoplasmic Ca^2+^ fluxes that relay the extracellular signal. Failure to remove excess intracellular Ca^2+^ from the cytoplasm or to restore presignal equilibrium dysregulates intracellular signaling and can lead to cell death (7, 8). As such, the mechanisms responsible for Ca^2+^ detoxification are of critical importance to eukaryotic cell viability.

In fungi, two systems are responsible for sequestering cytoplasmic Ca^2+^ ions into the vacuole, namely, the H^+^/Ca^2+^ exchanger Vcx1p (9–11), which uses the proton gradient across the vacuolar membrane (generated by the V-ATPase) to drive Ca^2+^ transport, and the P-type ATPase Pmc1p (11, 12). Both Pmc1p and Vcx1p are required for Cryptococcus neoformans to colonize lung or brain tissue in a mouse model of infection (13–15). While Aspergillus fumigatus, a major cause of pulmonary as well as disseminated infections of humans, has three Pmc1p homologues (*PMCA*, *PMCB*, and *PMCC*), with *PMCC* seemingly essential for viability and *PMCA* required for virulence in a neutropenic mouse model of invasive pulmonary aspergillosis (16). Ca^2+^-dependent signaling, particularly through the calcineurin signaling pathway, is also required for Candida albicans to tolerate the azole antifungals (17, 18). However, deletion of the *PMC1* gene has been reported to result in fluconazole resistance in C. albicans (18). The purpose of this study was to determine if Pmc1p or Vcx1p is required for C. albicans pathogenicity and how these pumps impact antifungal resistance.

## RESULTS

### Pmc1p is required for Candida albicans stress tolerance.

**FIG 1:**
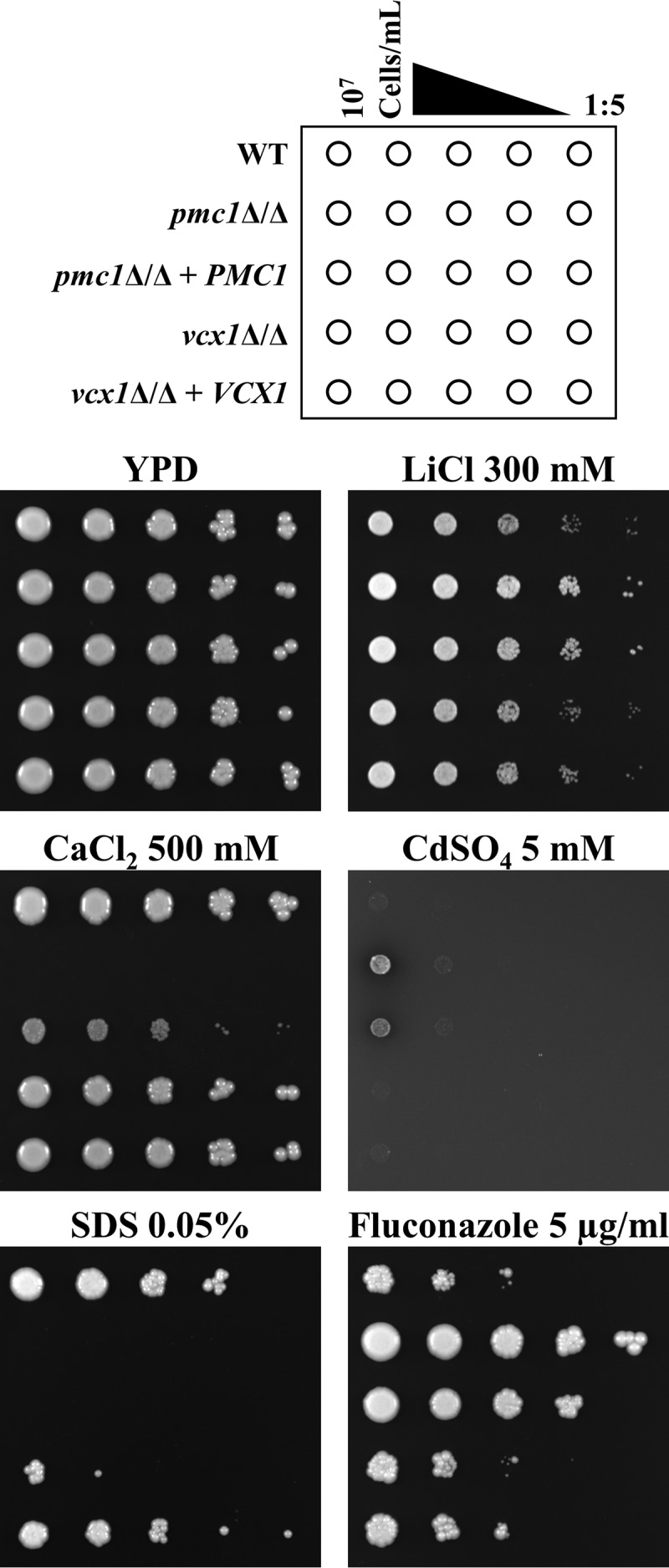
Candida albicans *pmc1*Δ/Δ mutant is hypersensitive to ionic and membrane stress. Wild-type (GP1), *pmc1*Δ/Δ, *vcx1*Δ/Δ, and revertant strains of C. albicans were suspended at 1 × 10^7^ cells/ml in sterile deionized water, and serial 1:5 dilutions were prepared. Cell suspensions were then applied to YPD agar plates or YPD agar supplemented with the indicated concentrations of LiCl, CaCl_2_, CdSO_4_, SDS, or fluconazole using a sterile multipronged applicator. Plates were incubated at 30°C for 48 to 96 h and then imaged.

To determine if Pmc1p or Vcx1p is required for C. albicans pathogenesis, we constructed *pmc1*Δ/Δ and *vcx1*Δ/Δ mutants using a PCR-based approach (19). Complemented strains were made by reintroducing a wild-type *PMC1* or *VCX1* allele into the *pmc1*Δ/Δ or *vcx1*Δ/Δ mutant, respectively, using an integrating vector that fully restores the *IRO1*-*URA3* locus. We initially examined phenotypes that have been associated with loss of Pmc1p or Vcx1p function in fungi (10–12, 15, 16, 18). Each of the described phenotypes was verified using at least two independently derived clones for each genotype. While the *pmc1*Δ/Δ and *vcx1*Δ/Δ mutants grew to a similar extent as the wild-type control strain on yeast extract-peptone-dextrose (YPD) agar plates, the *pmc1*Δ/Δ mutant was severely impaired by high concentrations of CaCl_2_ (Fig. 1A; see also Fig. S1 in the supplemental material). The *pmc1*Δ/Δ mutant was more resistant than the wild type to LiCl and CdSO_4_ but hypersensitive to the membrane stressor SDS (Fig. 1A). Reintroduction of *PMC1* into the *pmc1*Δ/Δ mutant only partially restored wild-type phenotypes with respect to LiCl, CaCl_2_, and CdSO_4_ sensitivity, suggesting that *PMC1* may be haploinsufficient. The *PMC1* reconstituted strain also remained completely sensitive to SDS at the concentration tested (0.05%), suggesting that the capacity of C. albicans to survive plasma membrane stress is especially sensitive to Pmc1p deficiencies. The growth of the *vcx1*Δ/Δ mutant, on the other hand, was unaffected by CaCl_2_, LiCl, or CdSO_4_ supplements (Fig. 1A). The *vcx1*Δ*/*Δ mutant was, however, sensitive to SDS, albeit to a lesser extent than the *pmc1*Δ/Δ mutant. These results confirm the importance of C. albicans Pmc1p for Ca^2+^ homeostasis and resistance to ionic as well as membrane stress.

10.1128/mSphere.00715-18.1 FIG S1 Growth of the Candida albicans *pmc1*Δ/Δ mutant is hypersensitive to high concentrations of CaCl_2_. Wild-type (GP1), *pmc1*Δ/Δ, *vcx1*Δ/Δ, and revertant strains were subcultured in YPD broth with or without 50 mM CaCl_2_ and incubated at 30°C for 24 h. Growth was measured at 30-min intervals by reading optical density at 600 nm (OD_600_). The mean and standard deviation from two biological replicates is shown for each condition and strain. Download FIG S1, DOCX file, 0.2 MB. Copyright © 2019 Luna-Tapia et al. 2019 Luna-Tapia et al. This content is distributed under the terms of the Creative Commons Attribution 4.0 International license.

### Pmc1p influences Candida albicans antifungal susceptibility.

A previous study reported that a C. albicans *pmc1*Δ/Δ mutant was resistant to the antifungal drug fluconazole (18). Similarly, we found our *pmc1Δ/Δ* mutant to be more resistant than the wild type to fluconazole when grown at 30°C on YPD agar supplemented with the drug (Fig. 1A) or using Etest strips (see Fig. S2). However, when tested in liquid RPMI medium at 35°C according to the standards of the CLSI protocol (20), or on RPMI agar with Etest strips (Fig. S2), the *pmc1Δ/Δ* mutant was as susceptible as the wild type. This indicated that the fluconazole resistance phenotype of the *pmc1Δ/Δ* mutant was dependent on the medium or some other growth condition. To determine how temperature influences the *pmc1*Δ/Δ mutants’ susceptibility to fluconazole, we compared the sensitivity of our C. albicans strains on YPD agar using spot dilution assays, following incubation at either 30, 35, 37, or 42°C. This revealed that while the *pmc1*Δ/Δ mutant is more resistant to fluconazole than the wild type at 30°C, it was not significantly different at 35 or 37°C and, paradoxically, was more sensitive at 42°C (see Fig. S3). Thus, the effect of Pmc1p on C. albicans sensitivity to fluconazole was temperature dependent. Notably, the *vcx1*Δ/Δ mutants’ sensitivity to fluconazole was indistinguishable from that of the wild type under all conditions tested.

10.1128/mSphere.00715-18.2 FIG S2 Candida albicans *pmc1Δ/Δ* mutant’s susceptibility to fluconazole is condition dependent. Cell suspensions of either wild-type (GP1), *pmc1*Δ/Δ, *vcx1*Δ/Δ, and the corresponding isogenic control strains of C. albicans were swabbed to the surface of either YPD or RPMI agar plates using cotton applicators, before a fluconazole Etest strip was placed in the center of the plate. Plates were imaged following incubation at 35°C for 24 h. Download FIG S2, DOCX file, 2.3 MB. Copyright © 2019 Luna-Tapia et al. 2019 Luna-Tapia et al. This content is distributed under the terms of the Creative Commons Attribution 4.0 International license.

10.1128/mSphere.00715-18.3 FIG S3 C. albicans *pmc1*Δ/Δ mutant’s response to azole treatment is temperature dependent. The susceptibilities of the C. albicans wild-type (GP1), *pmc1*Δ/Δ and *vcx1*Δ/Δ mutants, as well as their isogenic control strains to fluconazole were compared on YPD plates supplemented with 5 µg/ml of fluconazole. Cells were grown overnight in YPD at 30°C and resuspended at 1 × 10^7^ cells/ml suspension in sterile deionized water, and serial 1:5 dilutions were prepared. Cell suspensions were then applied to YPD agar plates or YPD agar plus fluconazole. Plates were incubated at 30°C, 35°C, 37°C, or 42°C for 48 h and then imaged. Download FIG S3, DOCX file, 0.6 MB. Copyright © 2019 Luna-Tapia et al. 2019 Luna-Tapia et al. This content is distributed under the terms of the Creative Commons Attribution 4.0 International license.

Interestingly, the *pmc1*Δ/Δ mutant was slightly more sensitive than the wild type to the morpholine antifungal amorolfine (see Fig. S4). Amorolfine inhibits both C-8 sterol isomerase (Erg2p) and C-14 sterol reductase (Erg24p), both of which act downstream of Erg11p in the ergosterol biosynthetic pathway (21). Again, the susceptibility of the *vcx1*Δ/Δ mutant to amorolfine was indistinguishable from that of the wild-type control (data not shown).

10.1128/mSphere.00715-18.4 FIG S4 Candida albicans *pmc1*Δ/Δ mutant is sensitive to amorolfine. Wild type (GP1), *pmc1*Δ/Δ mutant, and an isogenic revertant strain of C. albicans were grown in the presence of increasing concentrations of the morpholine antifungal amorolfine according to the CLSI broth microdilution protocol. After 48 h of incubation, growth was measured as OD_600_ and expressed as a percentage of the growth in the minus drug (DMSO alone) control wells. The means from three biological replicates are shown. Download FIG S4, DOCX file, 0.1 MB. Copyright © 2019 Luna-Tapia et al. 2019 Luna-Tapia et al. This content is distributed under the terms of the Creative Commons Attribution 4.0 International license.

### Loss of Pmc1p impairs Candida albicans hyphal formation.

**FIG 2:**
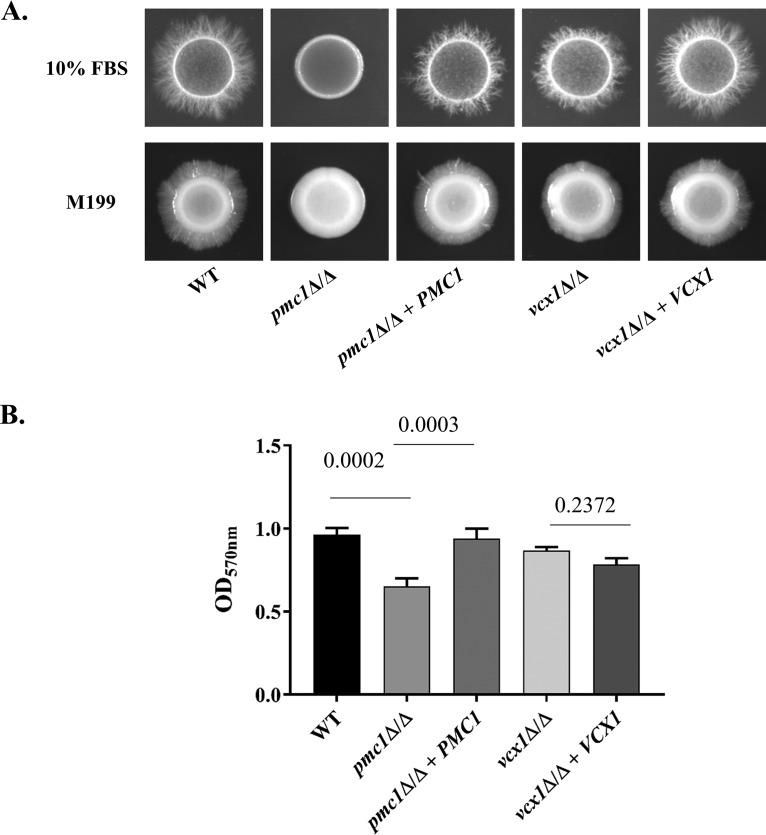
Candida albicans *pmc1*Δ/Δ mutant is defective in hyphal growth and biofilm formation. The abilities of the wild-type (GP1), *pmc1*Δ/Δ and *vcx1*Δ/Δ mutants, and isogenic control strains to form hyphae and biofilms were compared. (A) Each strain was resuspended at 1 × 10^7^ cells/ml in sterile deionized water, and 2.5 µl was spotted onto either M199 or 10% FBS agar plates. The resulting colonies were imaged after 96 h of incubation at 37°C. (B) Each strain was suspended at 1 × 10^6^ cells/ml in RPMI medium (pH 7.0), and 200 µl was dispensed into the wells of a flat-bottomed 96-well plate. After incubating at 37°C for 24 h, the plate was rinsed with PBS, and biofilm formation was detected using a crystal violet staining procedure. Crystal violet was subsequently eluted with 95% ethanol, and resolubilized dye was quantified by measuring the OD_570_ in a microplate reader. The means and standard errors of the means from three biological replicates are shown. The mean of each group was compared using a two-way analysis of variance (ANOVA), and *P* values of relevant comparisons are indicated.

**FIG 3:**
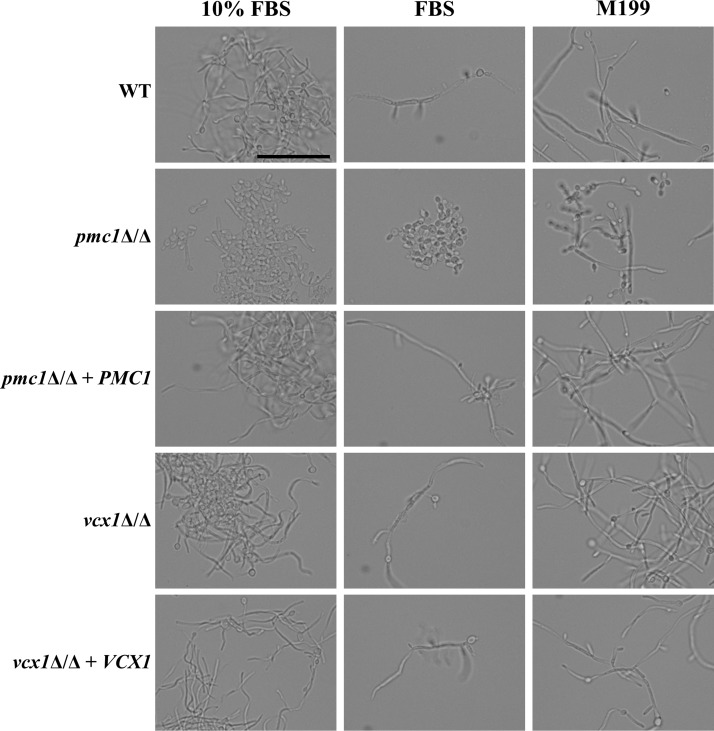
Candida albicans *pmc1*Δ/Δ mutant is defective in hyphal formation. Wild-type (GP1), *pmc1*Δ/Δ, *vcx1*Δ/Δ, and revertant strains of C. albicans were subcultured at ∼1 × 10^6^ cells/ml in 10% FBS, 100% FBS, or M199 and incubated at 37°C with shaking. Samples were taken after 6 h of incubation and fixed with formalin. Cell morphologies were observed by light microscopy using a 40× objective. Bar, 50 µm.

The ability to form hyphae is important for C. albicans pathogenicity (22, 23). We therefore examined the *pmc1*Δ/Δ and *vcx1*Δ/Δ mutants’ capacity to form hyphae. The *pmc1*Δ/Δ mutant’s ability to form hyphae was severely impaired on M199 or 10% fetal bovine serum (FBS) agar (Fig. 2A), and it remained as yeast cells in liquid FBS (Fig. 3). While a significant fraction of *pmc1*Δ/Δ cells produced short filaments in liquid M199 (Fig. 3), these were substantially shorter than for the wild type, and many cells remained in the yeast form under these conditions. In contrast, the *vcx1*Δ/Δ mutant exhibited no detectable defects in hyphal growth under any of these conditions, indicating that Vcx1p is not required for hyphal growth in C. albicans.

**FIG 4:**
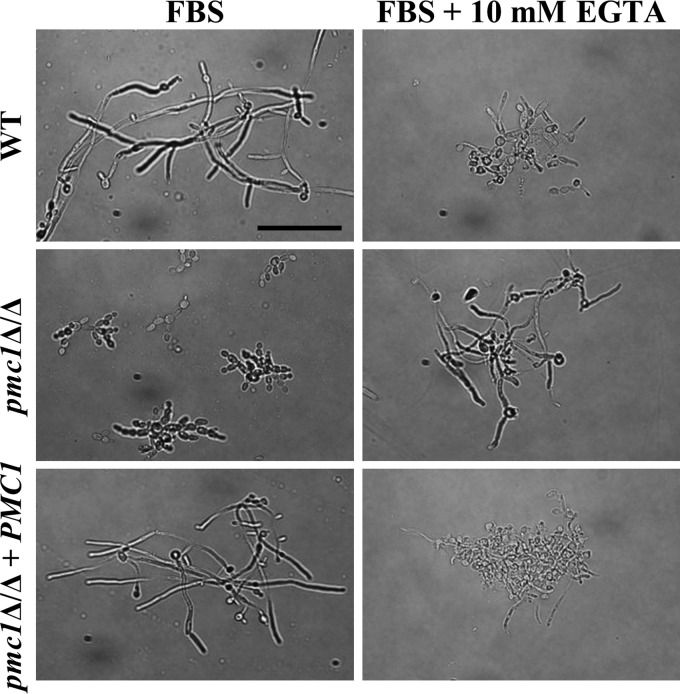
The Candida albicans *pmc1*Δ/Δ mutant’s hyphal growth defect is calcium dependent. Wild-type (GP1), *pmc1*Δ/Δ, and revertant strains of C. albicans were subcultured in FBS or FBS plus 10 mM EGTA at ∼1 × 10^6^ cells/ml and incubated at 37°C with shaking. Samples were taken after 6 h of incubation, and cells were fixed with formalin. Cell morphologies were subsequently observed by light microscopy using a 40× objective. Bar, 50 µm.

Since the *pmc1*Δ/Δ mutant was hypersensitive to high levels of Ca^2+^ and unable to form normal hyphae in FBS, we next evaluated if the defects in hyphal formation were due to the high levels of Ca^2+^ found in serum (3.5 to 4 mM) (24). This was tested using the Ca^2+^ chelator EGTA to sequester free Ca^2+^ in the FBS. The addition of 5 mM EGTA to the FBS largely restored the ability of the *pmc1*Δ/Δ mutant to form filaments (Fig. 4). Interestingly, the ability of the wild type and the complemented control strain to form hyphae was reduced by the addition of EGTA to the FBS. These results establish that the calcium concentrations within host tissues and fluids, as well as the calcium detoxification functions performed by Pmc1p, are an essential determinant of fungal morphogenesis and therefore C. albicans pathogenicity.

Finally, since hyphal growth is intimately linked to the capacity of C. albicans to form biofilms (25), we compared the *pmc1*Δ/Δ, *vcx1*Δ/Δ, and wild-type control strains abilities to form biofilms using a simple *in vitro* assay. Again, the *pmc1*Δ/Δ mutant was significantly impaired in its capacity to form biofilms, while the *vcx1*Δ/Δ mutant was able to form biofilms to the same extent as the wild-type control and complemented strains (Fig. 2B).

### Pmc1p is required for Candida albicans virulence in a mouse model of disseminated infection.

**FIG 5:**
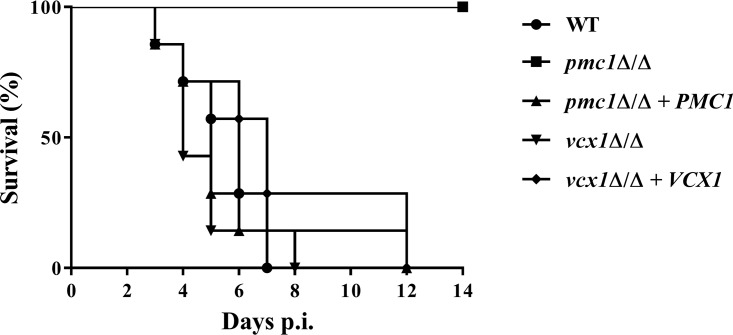
Candida albicans *pmc1Δ/Δ* mutant is avirulent in a mouse model of disseminated candidiasis. Groups of BALB/c mice (*N* = 7) were inoculated with ∼5 × 10^5^ CFU of either wild-type (GP1), *pmc1*Δ/Δ, *vcx1*Δ/Δ, or revertant strains via lateral tail vein injections. The mice were then monitored 3 times daily for 14 days, and those showing signs of distress were humanely euthanized. The survival of each group was compared using the log rank test (*P* < 0.0001).

To determine if either Pmc1p or Vcx1p is required for C. albicans to cause disease within its mammalian host, we compared the virulence of the *pmc1*Δ/Δ and *vcx1*Δ/Δ mutants with that of the wild type in a mouse model of disseminated infection (Fig. 5). All mice infected with wild-type C. albicans succumbed within 7 days of infection. However, all 7 of the mice infected with the *pmc1*Δ/Δ mutant survived the duration of the experiment (14 days), with 3 having undetectable levels of fungal colonization within their kidneys and the remainder having relatively low levels (ranging from 3.32 × 10^3^ to 2.34 × 10^4^ CFU/g of kidney). In contrast, the *vcx1*Δ/Δ mutant was as virulent as the wild-type and revertant control strains, as determined by the comparable survival times of mice infected with each strain. These data indicate that Pmc1p, but not Vcx1p, is required for C. albicans pathogenicity following dissemination through the bloodstream (Fig. 5).

## DISCUSSION

In this study, we explored the contributions of the Pmc1p Ca^2+^ pump and the Vcx1p Ca^2+^ exchanger to stress response and pathogenesis of C. albicans. It is evident from these results that the inability of the *pmc1*Δ/Δ mutant to properly remove Ca^2+^ from the cytoplasm into the vacuole renders the excess Ca^2+^ toxic to the mutant in a Ca^2+^-rich medium.

Another interesting finding was the impairment of hyphal formation in the *pmc1*Δ/Δ mutant in M199 medium and FBS. This suggests that the regulation of Ca^2+^ fluxes by Pmc1p into the vacuole is important for the yeast-to-hyphae transition and follows the findings of previous work indicating that Ca^2+^ release from the fungal vacuole into the cytoplasm via the Ca^2+^ channel Yvc1p is important for hyphal formation (26). This highlights the importance of Ca^2+^ uptake into and release from the fungal vacuole in morphogenesis. Interestingly, removal of free Ca^2+^ from the FBS allowed the *pmc1*Δ/Δ mutant to form filaments. Thus, while the concentration of calcium in FBS (∼3.5 to 4 mM) (24) was substantially lower than that used to test the calcium toxicity herein, it was nonetheless sufficient to impair the mutant’s capacity to form hyphae, a characteristic that is intimately associated with the ability of C. albicans to cause disease (22, 23). Our results further suggest that the *pmc1*Δ/Δ mutant does not have a mechanical defect in its ability to form hyphae, but rather its inability to remove excess Ca^2+^ from the cytoplasm interferes with the requisite signaling events. It is likely that the dysregulation of Ca^2+^-based signaling also underlies the wide range of physiological and stress-related phenotypes of the *pmc1*Δ/Δ mutant.

C. albicans mutants deficient in calcineurin signaling were previously reported as exquisitely sensitive to Ca^2+^ levels in serum (18, 24) and had severe defects in the colonization of kidney tissue in a mouse model of disseminated infection (18, 27). This makes sense, since Pmc1p is a downstream effector of calcineurin signaling (18, 28); therefore, a loss of Pmc1p would be expected to produce a similar phenotypic profile to that resulting from calcineurin dysfunction. Collectively, the phenotypic deficiencies of the *pmc1*Δ/Δ mutant resulting from its reduced capacity to sequester excess Ca^2+^ into the vacuole likely underlie its inability to colonize or invade mammalian tissue in the mouse model of disseminated infection. Interestingly, loss of Vcx1p function had little consequence on C. albicans physiology *in vitro* or pathogenicity *in vivo*. Given that Pmc1p is a low-capacity high-affinity Ca^2+^ transporter, while Vcx1p is a high-capacity low-affinity transporter, our results suggest that restoring a low cytoplasmic concentration of Ca^2+^ following a signaling event may be more important with respect to avoiding toxicity than the rate at which Ca^2+^ is initially removed.

While most Pmc1p homologs are not essential for fungal viability *in vitro*, their function appears to be essential for the survival of C. albicans, C. neoformans and A. fumigatus *in vivo*, i.e., within mammalian tissue (13, 15, 16). Furthermore, even partially suppressing the expression of either of two Pmc1p orthologues that localize to intracellular acidocalcisomes is sufficient to cause gross morphological abnormalities and severely inhibit the growth of Trypanosoma brucei (29), the causative agent of African sleeping sickness. Collectively, these data underscore the critical importance of vacuolar Ca^2+^ uptake by the high-affinity Pmc1p transporter in promoting the survival of infectious eukaryotes within the mammalian host. Given the severity of the pathogenesis defects that result from loss of Pmc1p activity in each of these human pathogens, and in particular, the consequences upon fungal colonization of mammalian tissue, Pmc1p could provide a potentially efficacious target for antifungal therapy. Alternative approaches that prevent the sequestration of Ca^2+^ within the fungal vacuole, or else mobilize intravacuolar calcium, leading to toxicity, may also be viable therapeutic strategies. Whether Pmc1p itself is vulnerable to small-molecule inhibition and if inhibitors with sufficient fungal selective activity can be derived to provide the basis of a viable antimicrobial pharmacotherapy remain to be determined.

Pmc1p is most closely related to the PMCA-type Ca^2+^ pumps of mammals but has several important distinctions (11, 30). First, fungal Pmc1p proteins localize to the vacuolar membrane rather than the plasma membrane, and thus sequester excess calcium into an intracellular store rather than out of the cell (11, 30). Second, while fungal Pmc1p has been classified as a type 2B P-type ATPase, which are typically characterized by a cytoplasmic autoregulatory domain, fungal Pmc1p proteins completely lack this domain. In mammals, the autoinhibitory domain of all four PMCA pumps is found within an extended cytoplasmic C-terminal domain, shown by crystallography to wrap around and block the catalytic core of the protein (31, 32). Intracellular Ca^2+^ fluxes activate PMCA following Ca^2+^-calmodulin binding to specific sequences in its C terminus that release autoinhibition. Curiously, fungal Pmc proteins completely lack extended N or C termini or the calmodulin binding sites that form the basis of the autoregulatory domains found in higher eukaryotes (11, 31–34). Thus, key structural determinants, as well as the molecular mechanisms by which the activity of these pumps are regulated in infectious fungi, are fundamentally different from those found in their mammalian host.

Previous reports, which were confirmed in this study, found that C. albicans *pmc1*Δ/Δ mutants are resistant to fluconazole on agar plates (18), while their susceptibility is not detectably different from that of the wild type when the standard CLSI broth microdilution protocol is used (20). Here, we determined that the *pmc1*Δ/Δ mutant’s reduced susceptibility to fluconazole was temperature dependent and therefore may relate to a form of azole tolerance known as “trailing growth” (35) rather than outright azole resistance. Trailing growth is observed with a significant proportion of C. albicans isolates and manifests as significant residual growth in the presence of the azole that becomes apparent after 48 h of incubation. In its extreme form, trailing growth can be mistaken for true azole resistance; however, patients and experimental animals infected with trailing isolates generally respond well to treatment with the azoles (36, 37). The effect of temperature on azole tolerance was also observed previously in an endosomal trafficking mutant lacking the Rab GTPase Vps21p. A C. albicans *vps21*Δ/Δ mutant displayed enhanced growth in the presence of fluconazole at 35°C but not at 30°C (38). This enhanced growth in the presence of fluconazole also resembled the trailing growth phenomenon and appeared to be dependent on elevated Ca^2+^ signaling (20, 38). Together, these findings further emphasize the importance of temperature and exogenous Ca^2+^ levels, as well as intracellular Ca^2+^ homeostasis, as determinants of the capacity of C. albicans to survive following exposure to the azole antifungals.

## MATERIALS AND METHODS

### Growth conditions.

C. albicans was routinely grown on YPD medium (1% yeast extract, 2% peptone, 2% dextrose) at 30°C, supplemented with uridine (50 μg/ml) when necessary. Transformant selection was carried out on minimal YNB medium (6.75 g/liter yeast nitrogen base without amino acids, 2% dextrose, 2% Bacto agar), supplemented with the appropriate auxotrophic requirements as described for Saccharomyces cerevisiae (39) or 50 μg/ml uridine.

### Plasmid construction.

All oligonucleotides used in this study are listed in Table S1 in the supplemental material.

10.1128/mSphere.00715-18.5 TABLE S1 List of oligonucleotides used in this study. Download Table S1, PDF file, 0.1 MB. Copyright © 2019 Luna-Tapia et al. 2019 Luna-Tapia et al. This content is distributed under the terms of the Creative Commons Attribution 4.0 International license.

The *PMC1* open reading frame (ORF) with 761 bp of 5′- and 434 nt of 3′-untranslated-region (UTR) sequences was amplified from SC5314 genomic DNA (gDNA) with primer pair PMC1AMPF-KpnI and PMC1AMPR-SacI and then cloned between the KpnI and SacI restriction sites of pLUX (43) to produce plasmid pLUXPMC1.

For construction of the plasmid pLUXVCX1, the *VCX1* ORF with 747 bp of 5′- and 324 bp of 3′-UTR sequences was amplified using primer pair VCX1AMPF-KpnI and VCX1AMPR-SacI and cloned between the KpnI and SacI restriction sites of pLUX.

### Candida albicans strains.

All strains used in this study are listed in Table S2. Transformation of C. albicans with DNA constructs was performed using the lithium acetate method (40). Gene deletion strains were constructed by the PCR-based approach described by Wilson et al. (19), using the *ura3*Δ/Δ*his1*Δ/Δ*arg4*Δ/Δ strain BWP17, kindly provided by Aaron Mitchell (Carnegie Mellon University).

10.1128/mSphere.00715-18.6 TABLE S2 List of strains used in this study. Download Table S2, PDF file, 0.1 MB. Copyright © 2019 Luna-Tapia et al. 2019 Luna-Tapia et al. This content is distributed under the terms of the Creative Commons Attribution 4.0 International license.

The *pmc1*Δ/Δ strain was constructed as follows. *PMC1* deletion cassettes were amplified by PCR with primers PMC1DISF and PMC1DISR using pRS-ARG4ΔSpeI, pGEM-HIS1, or pDDB57 (containing a recyclable *URA3*-*dpl200* marker) (20, 21) as the template. Each *PMC1* allele was then sequentially deleted from BWP17 using *HIS1* and *ARG4* markers to generate *pmc1*Δ/Δ *ura3*Δ/Δ gene deletion mutants. The correct integration of the deletion cassettes was confirmed at each step by PCR with the following primers sets: ARG4INTF2/PMC1AMPR-SacI and ARG4INTR2/PMC1AMPF-KpnI for *ARG4* integration or HIS1INTR2/PMC1AMPR-SacI and HIS1INTF2/PMC1AMPF-KpnI for *HIS1* integration. The absence of an intact *PMC1* allele was confirmed using primer pair PMC1DETF and PMC1DETR. Isogenic mutant and *PMC1*-reconstituted strains were produced by transforming the *pmc1*Δ/Δ *ura3*Δ/Δ mutant with either pLUX (vector alone) or pLUXPMC1 after digestion with NheI. The correct integration of the pLUX vector fully restores *URA3* and adjacent *IRO1* loci, and this was confirmed by PCR using primer pair LUXINTDETF and LUXINTDETR.

*VCX1* deletion cassettes were amplified by PCR with primers VCX1DISF and VCX1DISR using pRS-ARG4ΔSpeI, pGEM-HIS1, or pDDB57 as the templates. The *vcx1*Δ/Δ *ura3*Δ/Δ gene deletion mutants were produced by sequential deletion of each *VCX1* allele using *HIS1* and *ARG4* markers. Correct integration of deletion cassettes was confirmed at each step by PCR with primers pairs ARG4INTF2/VCX1AMPR-SacI and ARG4INTR2/VCX1AMPF-KpnI (*ARG4* integration), or HIS1INTR2/VCX1AMPR-SacI and HIS1INTF2/VCX1AMPF-KpnI (*HIS1* integration). Lack of an intact *VCX1* allele was confirmed by using primer pair VCX1DETF/VCX1DETR. Isogenic *vcx1*Δ/Δ mutant and *VCX1*-reconstituted strains were produced by transforming the *vcx1*Δ/Δ *ura3*Δ/Δ mutant with either NheI-digested pLUX (vector alone) or pLUXVCX1. Correct integration of either plasmid was confirmed by PCR using primer pair LUXINTDETF/LUXINTDETR.

### Stress resistance and hyphal growth assays.

Each C. albicans strain was grown overnight in YPD at 30°C. The cells were washed in sterile deionized water, the cell density was adjusted to 10^7^ cells/ml, and 1:5 serial dilutions were performed in a 96-well plate. Each cell suspension was then applied to agar plates using a sterile multipronged applicator. Resistance to different stresses was determined on YPD agar containing 5 µg/ml of fluconazole, 0.05% SDS, 1.5 M NaCl, 300 mM LiCl, 5 mM CdSO_4_, or 500 mM CaCl_2_, with incubation at 30°C for 48 to 96 h. To induce hyphal growth, for each strain, 2.5 µl of a 10^7^ cells/ml cell suspension was spotted on M199 or 10% fetal bovine serum (FBS) agar plates, and incubated for 96 h at 37°C.

### Biofilm formation assay.

Biofilm formation was assessed using a protocol based on that described by O’Toole (41). Each C. albicans strain was grown overnight in YPD broth at 30°C. Each culture was then washed two times in sterile phosphate-buffered saline (PBS), the cell density was adjusted to 1 × 10^6^ per ml in RPMI medium (pH 7.0), and 200 µl was dispensed into the wells of a flat-bottomed 96-well plate. After incubation at 37°C for 24 h, each well was rinsed 3 times with sterile PBS and then stained with 0.01% crystal violet for 15 min. Each well was again rinsed 3 times with sterile water, and the dye was eluted with 95% ethanol (200 μl/well); 150 μl of resolubilized dye from each well was then transferred to a new flat-bottomed microtiter plate, and the optical density at 570 nm (OD_570_) was measured using a microplate reader.

### Antifungal susceptibility testing.

Antifungal susceptibility testing of all the strains included in this study was performed using the broth microdilution method described in the CLSI document M27-A3 (42) in a 96-well plate format. All drugs for susceptibility testing used in this study were diluted in dimethyl sulfoxide (DMSO) to 200 times the final concentration. RPMI 1640 medium (Sigma-Aldrich) was prepared according to the CLSI document; the medium was buffered with morpholinepropanesulfonic acid (MOPS) and pH adjusted using NaOH and HCl. Plates were incubated at 25°C, 35°C, or 42°C without shaking for 24 or 48 h. The content of each well was carefully resuspended by pipetting up and down before the OD_600_ was measured using a Biotek Cytation 5 plate reader.

Susceptibility testing using fluconazole Etest strips was performed on agar plates with MOPS-buffered RPMI medium at pH 7. Cell suspensions were streaked onto the RPMI plates using sterile cotton applicators. Etest strips were applied on the surface of the agar, and the plates were incubated at 35°C for 24 to 48 h.

### Ethics statement.

The animals used in this study were housed in American Association for Accreditation of Laboratory Animal Care (AAALAC)-approved facilities at the University of Tennessee Health Science Center (UTHSC). The Institutional Animal Care and Use Committee (IACUC) at UTHSC approved the use of all animals and procedures (IACUC protocol numbers 15-081 and 16-156). Mice were given standard rodent chow and water *ad libitum*. Mice were monitored daily for signs of distress, including noticeable weight loss and lethargy, and for the body condition score. The IACUC at UTHSC uses the Public Health Policy on Humane Care and Use of Laboratory Animals (PHS) and the Guide for the Care and Use of Laboratory Animals as a basis for establishing and maintaining an institutional program for activities involving animals. To ensure high standards for animal welfare, the IACUC at UTHSC remains compliant with all applicable provisions of the Animal Welfare Act (AWAR), guidance from the Office of Laboratory Animal Welfare (OLAW), and the American Veterinary Medical Association Guidelines on Euthanasia.

### Mouse model of disseminated candidiasis.

C. albicans strains were grown overnight in YPD broth at 30°C with shaking. Stationary-phase cultures of C. albicans strains were washed twice in sterile, endotoxin-free phosphate-buffered saline (PBS) and resuspended in PBS at 5 × 10^6^ cells/ml. Groups of 6 BALB/c mice per C. albicans strain were then inoculated via tail vein injections with 100 μl of the desired cell suspension (∼5 × 10^5^ cells). Viable cell counts of each inoculum were confirmed by plating appropriate dilutions on YPD agar plates and counting the colonies formed after 48 h of incubation at 30°C. Mice were then monitored for 14 days postinfection, and those showing signs of distress were humanely euthanized. Animals surviving to the end of the experiment (day 14) were euthanized, and their kidneys were extracted, weighed, and homogenized in PBS. Serial dilutions of kidney homogenates were plated on YPD agar plates containing 50 μg/ml of chloramphenicol. The CFU/g of kidney tissue was then determined from the number of colonies formed on the plates after 48 h of incubation at 30°C.

## References

[1] Klionsky DJ, Herman PK, Emr SD 1990 The fungal vacuole: composition, function, and biogenesis. Microbiol Rev 54:266–292. 2215422 10.1128/mr.54.3.266-292.1990 PMC372777

[2] Veses V, Richards A, Gow NA 2008 Vacuoles and fungal biology. Curr Opin Microbiol 11:503–510. doi:10.1016/j.mib.2008.09.017. 18935977

[3] Cyert MS, Philpott CC 2013 Regulation of cation balance in *Saccharomyces cerevisiae*. Genetics 193:677–713. doi:10.1534/genetics.112.147207. 23463800 PMC3583992

[4] Dunn T, Gable K, Beeler T 1994 Regulation of cellular Ca^2+^ by yeast vacuoles. J Biol Chem 269:7273–7278. 8125940

[5] Bonilla M, Nastase KK, Cunningham KW 2002 Essential role of calcineurin in response to endoplasmic reticulum stress. EMBO J 21:2343–2353. doi:10.1093/emboj/21.10.2343. 12006487 PMC126012

[6] Kraus PR, Heitman J 2003 Coping with stress: calmodulin and calcineurin in model and pathogenic fungi. Biochem Biophys Res Commun 311:1151–1157. 14623301 10.1016/s0006-291x(03)01528-6

[7] Marchi S, Patergnani S, Missiroli S, Morciano G, Rimessi A, Wieckowski MR, Giorgi C, Pinton P 2018 Mitochondrial and endoplasmic reticulum calcium homeostasis and cell death. Cell Calcium 69:62–72. doi:10.1016/j.ceca.2017.05.003. 28515000

[8] Pinton P, Giorgi C, Siviero R, Zecchini E, Rizzuto R 2008 Calcium and apoptosis: ER-mitochondria Ca^2+^ transfer in the control of apoptosis. Oncogene 27:6407–6418. doi:10.1038/onc.2008.308. 18955969 PMC2844952

[9] Miseta A, Kellermayer R, Aiello DP, Fu L, Bedwell DM 1999 The vacuolar Ca^2+^/H^+^ exchanger Vcx1p/Hum1p tightly controls cytosolic Ca^2+^ levels in *S. cerevisiae*. FEBS Lett 451:132–136. 10371152 10.1016/s0014-5793(99)00519-0

[10] Cunningham KW, Fink GR 1996 Calcineurin inhibits VCX1-dependent H^+^/Ca^2+^ exchange and induces Ca^2+^ ATPases in *Saccharomyces cerevisiae*. Mol Cell Biol 16:2226–2237. 8628289 10.1128/mcb.16.5.2226 PMC231210

[11] Pittman JK 2011 Vacuolar Ca^2+^ uptake. Cell Calcium 50:139–146. doi:10.1016/j.ceca.2011.01.004. 21310481

[12] Cunningham KW, Fink GR 1994 Calcineurin-dependent growth control in *Saccharomyces cerevisiae* mutants lacking PMC1, a homolog of plasma membrane Ca^2+^ ATPases. J Cell Biol 124:351–363. 7507493 10.1083/jcb.124.3.351 PMC2119937

[13] Squizani ED, Oliveira NK, Reuwsaat JCV, Marques BM, Lopes W, Gerber AL, Vasconcelos Atr d, Lev S, Djordjevic JT, Schrank A, Vainstein MH, Staats CC, Kmetzsch L 2018 Cryptococcal dissemination to the central nervous system requires the vacuolar calcium transporter Pmc1. Cell Microbiol 20:e12803. doi:10.1111/cmi.12803. 29113016

[14] Kmetzsch L, Staats CC, Simon E, Fonseca FL, de Oliveira DL, Sobrino L, Rodrigues J, Leal AL, Nimrichter L, Rodrigues ML, Schrank A, Vainstein MH 2010 The vacuolar Ca^2+^ exchanger Vcx1 is involved in calcineurin-dependent Ca^2+^ tolerance and virulence in *Cryptococcus neoformans*. Eukaryot Cell 9:1798–1805. doi:10.1128/EC.00114-10. 20889719 PMC2976299

[15] Kmetzsch L, Staats CC, Cupertino JB, Fonseca FL, Rodrigues ML, Schrank A, Vainstein MH 2013 The calcium transporter Pmc1 provides Ca^2+^ tolerance and influences the progression of murine cryptococcal infection. FEBS J 280:4853–4864. doi:10.1111/febs.12458. 23895559

[16] Dinamarco TM, Freitas FZ, Almeida RS, Brown NA, dos Reis TF, Ramalho LN, Savoldi M, Goldman MH, Bertolini MC, Goldman GH 2012 Functional characterization of an *Aspergillus fumigatus* calcium transporter (PmcA) that is essential for fungal infection. PLoS One 7:e37591. doi:10.1371/journal.pone.0037591. 22649543 PMC3359301

[17] Cruz MC, Goldstein AL, Blankenship JR, Del Poeta M, Davis D, Cardenas ME, Perfect JR, McCusker JH, Heitman J 2002 Calcineurin is essential for survival during membrane stress in *Candida albicans*. EMBO J 21:546–559. 11847103 10.1093/emboj/21.4.546 PMC125859

[18] Sanglard D, Ischer F, Marchetti O, Entenza J, Bille J 2003 Calcineurin A of *Candida albicans*: involvement in antifungal tolerance, cell morphogenesis and virulence. Mol Microbiol 48:959–976. 12753189 10.1046/j.1365-2958.2003.03495.x

[19] Wilson RB, Davis D, Mitchell AP 1996 Rapid hypothesis testing with *Candida albicans* through gene disruption with short homology regions. J Bacteriol 181:1868–1874. 10.1128/jb.181.6.1868-1874.1999 PMC93587 10074081

[20] Luna-Tapia A, Tournu H, Peters TL, Palmer GE 2016 Endosomal trafficking defects can induce calcium dependent azole tolerance in *Candida albicans*. Antimicrob Agents Chemother 60:7170–7177. doi:10.1128/AAC.01034-16. 27645241 PMC5118996

[21] Barrett-Bee K, Dixon G 1995 Ergosterol biosynthesis inhibition: a target for antifungal agents. Acta Biochim Pol 42:465–479. 8852337

[22] Saville SP, Lazzell AL, Monteagudo C, Lopez-Ribot JL 2003 Engineered control of cell morphology *in vivo* reveals distinct roles for yeast and filamentous forms of *Candida albicans* during infection. Eukaryot Cell 2:1053–1060. 14555488 10.1128/EC.2.5.1053-1060.2003 PMC219382

[23] Lo HJ, Kohler JR, DiDomenico B, Loebenberg D, Cacciapuoti A, Fink GR 1997 Nonfilamentous *C. albicans* mutants are avirulent. Cell 90:939–949. 9298905 10.1016/s0092-8674(00)80358-x

[24] Blankenship JR, Heitman J 2005 Calcineurin is required for *Candida albicans* to survive calcium stress in serum. Infect Immun 73:5767–5774. doi:10.1128/IAI.73.9.5767-5774.2005. 16113294 PMC1231066

[25] Nobile CJ, Johnson AD 2015 *Candida albicans* biofilms and human disease. Annu Rev Microbiol 69:71–92. doi:10.1146/annurev-micro-091014-104330. 26488273 PMC4930275

[26] Yu Q, Wang F, Zhao Q, Chen J, Zhang B, Ding X, Wang H, Yang B, Lu G, Zhang B, Li M 2014 A novel role of the vacuolar calcium channel Yvc1 in stress response, morphogenesis and pathogenicity of *Candida albicans*. Int J Med Microbiol 304:339–350. doi:10.1016/j.ijmm.2013.11.022. 24368068

[27] Blankenship JR, Wormley FL, Boyce MK, Schell WA, Filler SG, Perfect JR, Heitman J 2003 Calcineurin is essential for *Candida albicans* survival in serum and virulence. Eukaryot Cell 2:422–430. 12796287 10.1128/EC.2.3.422-430.2003 PMC161442

[28] Stathopoulos AM, Cyert MS 1997 Calcineurin acts through the CRZ1/TCN1-encoded transcription factor to regulate gene expression in yeast. Genes Dev 11:3432–3444. 9407035 10.1101/gad.11.24.3432 PMC316814

[29] Luo S, Rohloff P, Cox J, Uyemura SA, Docampo R 2004 Trypanosoma brucei plasma membrane-type Ca^2+^-ATPase 1 (TbPMC1) and 2 (TbPMC2) genes encode functional Ca^2+^-ATPases localized to the acidocalcisomes and plasma membrane, and essential for Ca^2+^ homeostasis and growth. J Biol Chem 279:14427–14439. doi:10.1074/jbc.M309978200. 14724285

[30] Cunningham KW 2011 Acidic calcium stores of *Saccharomyces cerevisiae*. Cell Calcium 50:129–138. doi:10.1016/j.ceca.2011.01.010. 21377728 PMC3137693

[31] Di Leva F, Domi T, Fedrizzi L, Lim D, Carafoli E 2008 The plasma membrane Ca^2+^ ATPase of animal cells: structure, function and regulation. Arch Biochem Biophys 476:65–74. doi:10.1016/j.abb.2008.02.026. 18328800

[32] Enyedi A, Vorherr T, James P, McCormick DJ, Filoteo AG, Carafoli E, Penniston JT 1989 The calmodulin binding domain of the plasma membrane Ca^2+^ pump interacts both with calmodulin and with another part of the pump. J Biol Chem 264:12313–12321. 2526124

[33] Palmgren MG, Axelsen KB 1998 Evolution of P-type ATPases. Biochim Biophys Acta 1365:37–45. 9693719 10.1016/s0005-2728(98)00041-3

[34] Hofmann F, James P, Vorherr T, Carafoli E 1993 The C-terminal domain of the plasma membrane Ca^2+^ pump contains three high affinity Ca^2+^ binding sites. J Biol Chem 268:10252–10259. 8387515

[35] Arthington-Skaggs BA, Lee-Yang W, Ciblak MA, Frade JP, Brandt ME, Hajjeh RA, Harrison LH, Sofair AN, Warnock DW, Candidemia Active Surveillance Group. 2002 Comparison of visual and spectrophotometric methods of broth microdilution MIC end point determination and evaluation of a sterol quantitation method for *in vitro* susceptibility testing of fluconazole and itraconazole against trailing and nontrailing *Candida* isolates. Antimicrob Agents Chemother 46:2477–2481. 12121921 10.1128/AAC.46.8.2477-2481.2002 PMC127334

[36] Peters BM, Luna-Tapia A, Tournu H, Rybak JM, Rogers PD, Palmer GE 2017 An azole-tolerant endosomal trafficking mutant of *Candida albicans* is susceptible to azole treatment in a mouse model of vaginal candidiasis. Antimicrob Agents Chemother 61:e00084-17. doi:10.1128/AAC.00084-17. 28348159 PMC5444139

[37] Rex JH, Nelson PW, Paetznick VL, Lozano-Chiu M, Espinel-Ingroff A, Anaissie EJ 1998 Optimizing the correlation between results of testing *in vitro* and therapeutic outcome *in vivo* for fluconazole by testing critical isolates in a murine model of invasive candidiasis. Antimicrob Agents Chemother 42:129–134. doi:10.1128/AAC.42.1.129. 9449272 PMC105467

[38] Luna-Tapia A, Kerns ME, Eberle KE, Jursic BS, Palmer GE 2015 Trafficking through the late endosome significantly impacts *Candida albicans* tolerance of the azole antifungals. Antimicrob Agents Chemother 59:2410–2420. doi:10.1128/AAC.04239-14. 25666149 PMC4356793

[39] Burke D, Dawson D, Stearns T, Laboratory CSH 2000 Methods in yeast genetics: a cold spring harbor laboratory course manual. Cold Spring Harbor Laboratory Press, Cold Spring Harbor, NY.

[40] Gietz D, St Jean A, Woods RA, Schiestl RH 1992 Improved method for high efficiency transformation of intact yeast cells. Nucleic Acids Res 20:1425. 1561104 10.1093/nar/20.6.1425 PMC312198

[41] O'Toole GA 2011 Microtiter dish biofilm formation assay. J Vis Exp 30:2437. doi:10.3791/2437. PMC3182663 21307833

[42] Clinical and Laboratory Standards Institute. 2008 Reference method for broth dilution antifungal susceptibility testing of yeasts; approved standard, 3rd ed CLSI document M27-A3. Clinical and Laboratory Standards Institute, Wayne, PA.

[43] Ramon AM, Fonzi WA 2003 Diverged binding specificity of Rim101p, the *Candida albicans* ortholog of PacC. Eukaryot Cell 2:718–728. 12912891 10.1128/EC.2.4.718-728.2003 PMC178344

