# Supplementary figures and images for: The Vacuolar Ca2+ ATPase Pump Pmc1p Is Required for Candida albicans Pathogenesis
Source: mSphere. 2019 Feb 6;4(1):e00715-18. doi: 10.1128/mSphere.00715-18 (PMC6365616; doi:10.1128/mSphere.00715-18)

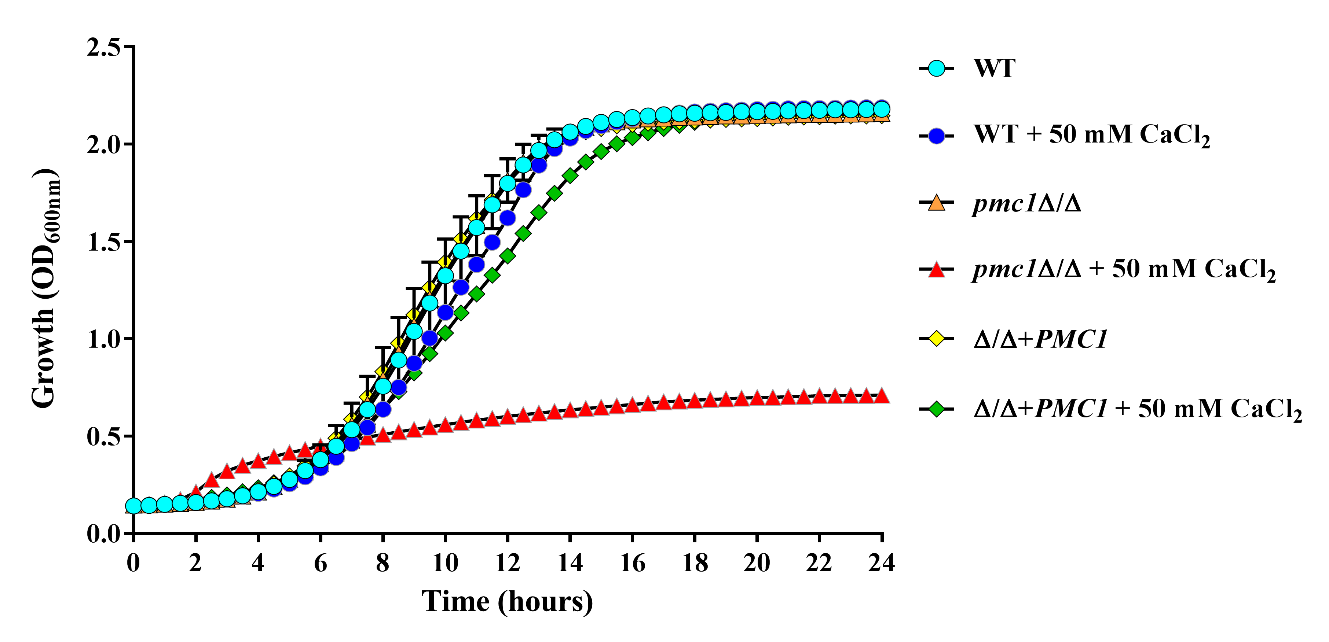


**Fig. S1.**

Supplement: FIG S1 [file mSphere.00715-18-sf001.docx]

**Fig. S2.**


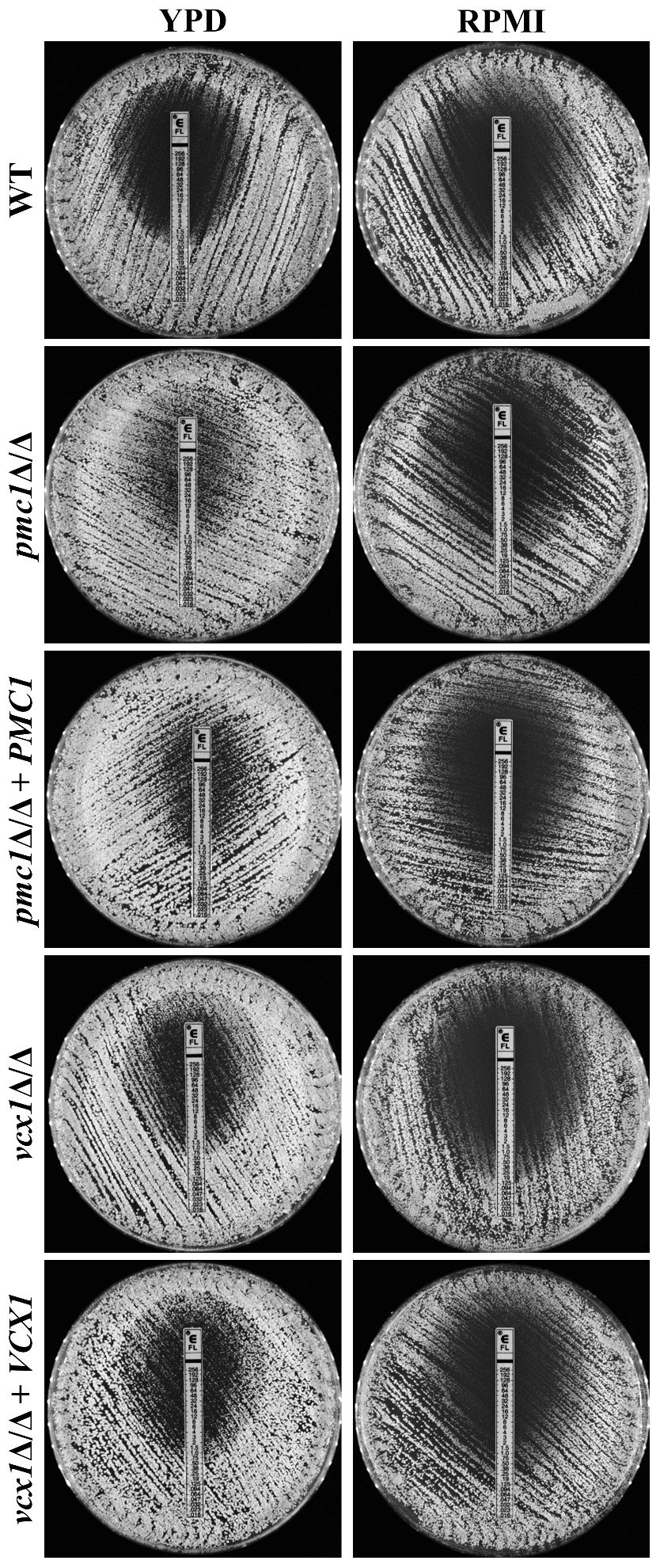

Supplement: FIG S2 [file mSphere.00715-18-sf002.docx]

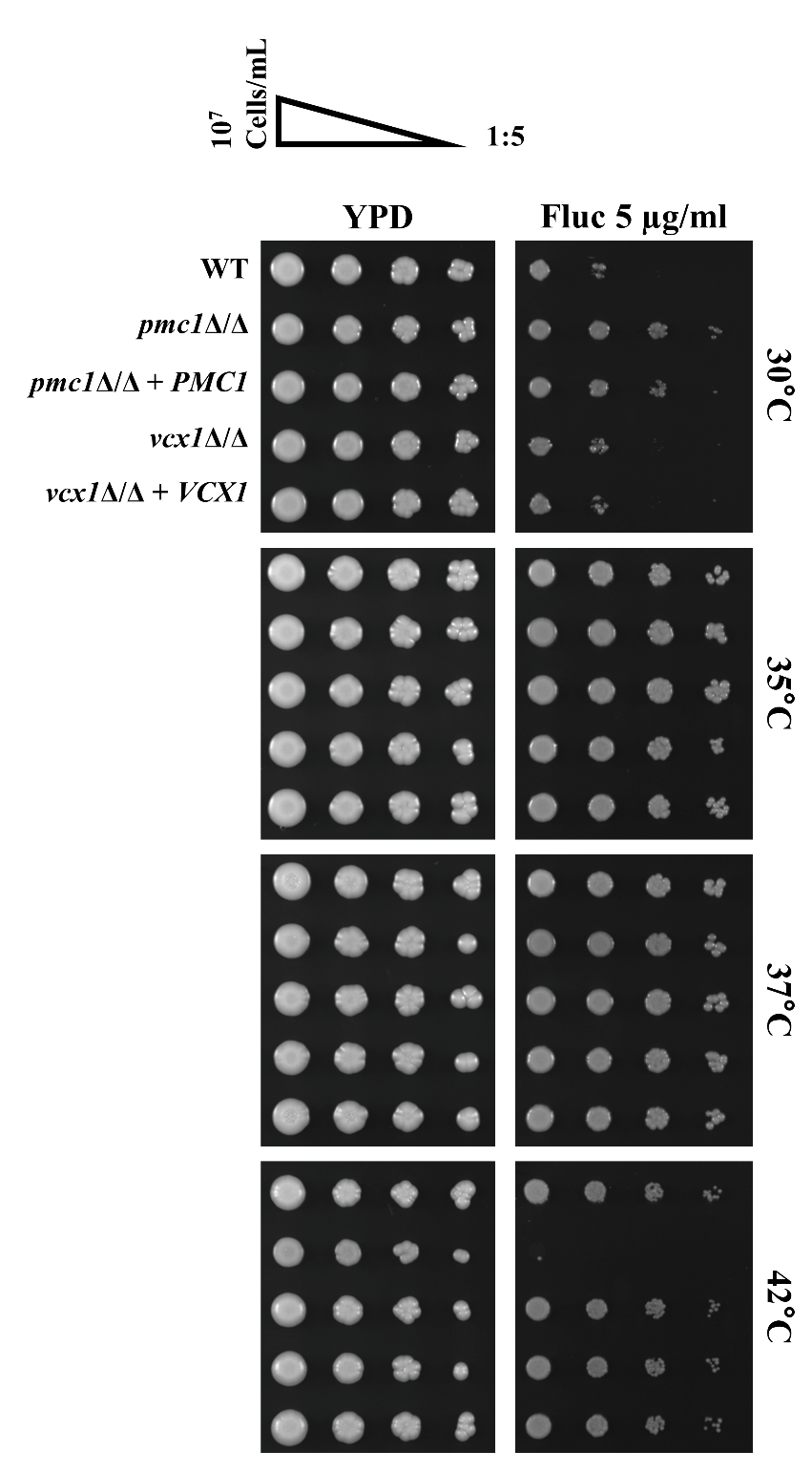


**Fig. S3.**

Supplement: FIG S3 [file mSphere.00715-18-sf003.docx]

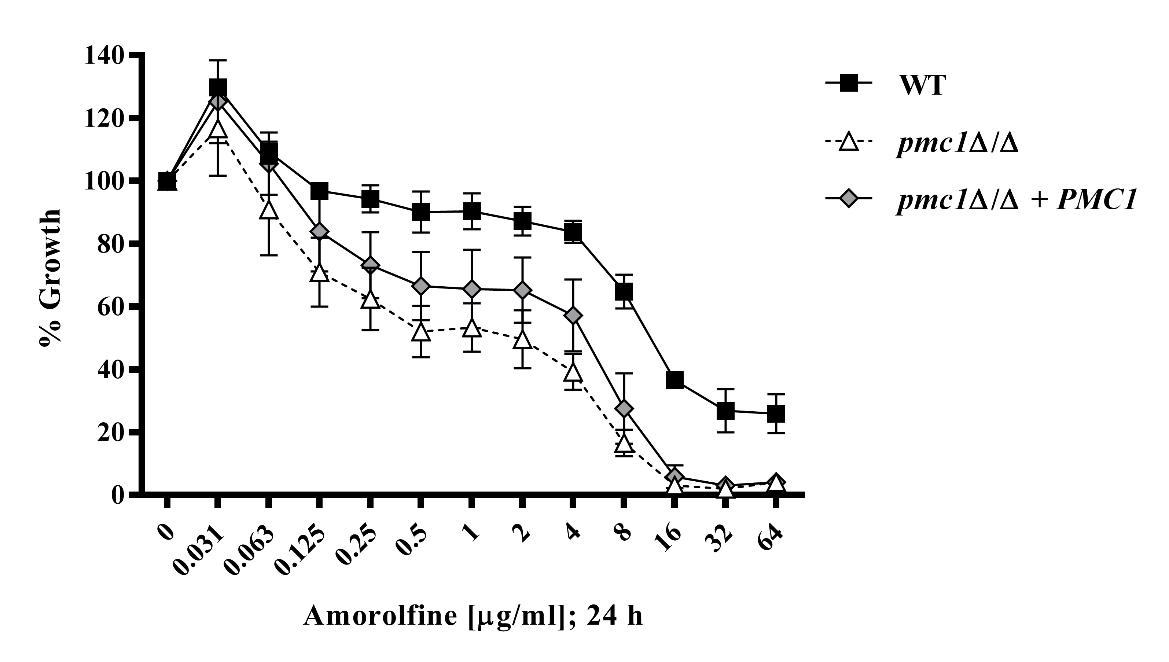


**Fig. S4.**

Supplement: FIG S4 [file mSphere.00715-18-sf004.docx]
