# Supplementary material for: The Vacuolar Ca2+ ATPase Pump Pmc1p Is Required for Candida albicans Pathogenesis
Source: mSphere. 2019 Feb 6;4(1):e00715-18. doi: 10.1128/mSphere.00715-18 (PMC6365616; doi:10.1128/mSphere.00715-18)
Supplement: TABLE S1 [file mSphere.00715-18-st001.pdf]

Table S1

PRIMER NAME	SEQUENCE (5'-3')
**ARG4INTF2**	AAGCTAGTGTGGAAAGAAGAG
**ARG4INTR2**	AATGACTGAATTATGTCGGTC
**HIS1INTF2**	ACTGTATCCTCTTCTGTCCCC
**HIS1INTR2**	CGACCATATGGGAGAGCTCCC
**LUXINTDETF**	CTGACCTTTAGTCTTTCCTGC
**LUXINTDETR**	CAGTAGTACTTGTTGTTGTATCG
**PMC1AMPF-KpnI**	TCA**<u>GGTACC</u>**GGTTCAATAAATTCCAATAGC
**PMC1AMPR-SacI**	TCA**<u>GAGCTC</u>**ATAACTACTATTACTTATTACTGG
**PMC1DETF**	CGGAAACAGTATTTTGGTATC
**PMC1DETR**	CACGTTTACATTGTAATACGGC
**PMC1DISF**	CCTTTCCAAAGCAAAGCAAAATTTTGAGTTCATATATACAAATTAAACATTATTACTTATCCTCATATTT*TGTGGAATTGTGAGCGGATA*
**PMC1DISR**	CCACTTTTAATCTATTCCTTTGATTCTTTTAAATCGGAATTACCACCACCTTCTAAATCCATTTCATCAT*GTTTTCCCAGTCACGACGTT*
**VCX1AMPF-KpnI**	TCA**<u>GGTACC</u>**CATCTTGATCTCCATAGGACC
**VCX1AMPR-SacI**	TCA**<u>GAGCTC</u>**TGTTGATCACTGACCAGTTGC
**VCX1DETF**	CTGGGGAATTAGAATGGAGTGC
**VCX1DETR**	CAACAATCTCATCAATTGATCC
**VCX1DISF**	ACTTTTCTATTATTCTACCTTGAATTCAAAGAATCCAAATTAAACAACAAATAAAACAAACCAACAGAGA*TGTGGAATTGTGAGCGGATA*
**VCX1DISR**	TACTCCTTTTTCAGCAAATATTCTATCATCTTCTCTTTCTTCCTCTTTCTTATCATCATGATAGTTGAAT*GTTTTCCCAGTCACGACGTT*

* Engineered restriction enzyme sites are highlighted in bold text and underlined. In italics, regions of homology for amplification of deletion cassettes.
