# Supplementary material for: The Vacuolar Ca2+ ATPase Pump Pmc1p Is Required for Candida albicans Pathogenesis
Source: mSphere. 2019 Feb 6;4(1):e00715-18. doi: 10.1128/mSphere.00715-18 (PMC6365616; doi:10.1128/mSphere.00715-18)
Supplement: TABLE S2 [file mSphere.00715-18-st002.pdf]

Supplemental Table S2

Strain	Relevant Genotype	Reference
BWP17	*ura3Δ/Δ his1Δ/Δ arg4Δ/Δ*	1
GP1 (“Wild-type”)	*ura3Δ/Δ:URA3 his1Δ/Δ:HIS1 arg4Δ/Δ:ARG4*	2
pmc1Δ/Δ	*ura3Δ/Δ:URA3 his1Δ/Δ arg4Δ/Δ pmc1Δ:HIS1/pmc1Δ:ARG4*	This study
pmc1Δ/Δ + PMC1	*ura3Δ/Δ:URA3: PMC1 his1Δ/Δ arg4Δ/Δ pmc1Δ:HIS1/pmc1Δ:ARG4*	This study
vcx1Δ/Δ	*ura3Δ/Δ:URA3 his1Δ/Δ arg4Δ/Δ vcx1Δ:HIS1/vcx1Δ:ARG4*	This study
vcx1Δ/Δ + VCX1	*ura3Δ/Δ:URA3: VCX1 his1Δ/Δ arg4Δ/Δ vcx1Δ:HIS1/vcx1Δ:ARG4*	This study

1. Wilson RB, Davis D, Mitchell AP. Rapid hypothesis testing with *Candida albicans* through gene disruption with short homology regions. Journal of Bacteriology. 1996;181(6):1868 - 74.
2. Luna-Tapia A, Willems HME, Parker JE, Tournu H, Barker KS, Nishimoto AT, et al. Loss of Upc2p-Inducible *ERG3* Transcription Is Sufficient To Confer Niche-Specific Azole Resistance without Compromising *Candida albicans* Pathogenicity. mBio. 2018;9(3):e00225-18.
